# Direct Numerical Simulation of Head-On Quenching of Statistically Planar Turbulent Premixed Methane-Air Flames Using a Detailed Chemical Mechanism

**DOI:** 10.1007/s10494-018-9907-5

**Published:** 2018-04-12

**Authors:** Jiawei Lai, Markus Klein, Nilanjan Chakraborty

**Affiliations:** 1 School of Engineering, Newcastle University Claremont Road, Newcastle-Upon-Tyne, NE1 7U UK; 2 Department of Aerospace Engineering, Universität der Bundeswehr München, Neubiberg, 85577 Germany

**Keywords:** Flame-wall interaction, Head-on quenching, Direct numerical simulation, Wall heat flux, Peclet number, Flame surface density, Scalar dissipation rate

## Abstract

A three-dimensional compressible Direct Numerical Simulation (DNS) analysis has been carried out for head-on quenching of a statistically planar stoichiometric methane-air flame by an isothermal inert wall. A multi-step chemical mechanism for methane-air combustion is used for the purpose of detailed chemistry DNS. For head-on quenching of stoichiometric methane-air flames, the mass fractions of major reactant species such as methane and oxygen tend to vanish at the wall during flame quenching. The absence of $\text {OH}$ at the wall gives rise to accumulation of carbon monoxide during flame quenching because $\text {CO}$ cannot be oxidised anymore. Furthermore, it has been found that low-temperature reactions give rise to accumulation of $\text {HO}_{2}$ and $\mathrm {H}_{2}\mathrm {O}_{2}$ at the wall during flame quenching. Moreover, these low temperature reactions are responsible for non-zero heat release rate at the wall during flame-wall interaction. In order to perform an in-depth comparison between simple and detailed chemistry DNS results, a corresponding simulation has been carried out for the same turbulence parameters for a representative single-step Arrhenius type irreversible chemical mechanism. In the corresponding simple chemistry simulation, heat release rate vanishes once the flame reaches a threshold distance from the wall. The distributions of reaction progress variable *c* and non-dimensional temperature *T* are found to be identical to each other away from the wall for the simple chemistry simulation but this equality does not hold during head-on quenching. The inequality between *c* (defined based on $\text {CH}_{4}$ mass fraction) and *T* holds both away from and close to the wall for the detailed chemistry simulation but it becomes particularly prominent in the near-wall region. The temporal evolutions of wall heat flux and wall Peclet number (i.e. normalised wall-normal distance of $T = 0.9$ isosurface) for both simple and detailed chemistry laminar and turbulent cases have been found to be qualitatively similar. However, small differences have been observed in the numerical values of the maximum normalised wall heat flux magnitude $\left ({\Phi }_{\max } \right )_{\mathrm {L}}$ and the minimum Peclet number $(Pe_{\min })_{\mathrm {L}}$ obtained from simple and detailed chemistry based laminar head-on quenching calculations. Detailed explanations have been provided for the observed differences in behaviours of $\left ({\Phi }_{\max }\right )_{\mathrm {L}}$ and $(Pe_{\min })_{\mathrm {L}}$. The usual Flame Surface Density (FSD) and scalar dissipation rate (SDR) based reaction rate closures do not adequately predict the mean reaction rate of reaction progress variable in the near-wall region for both simple and detailed chemistry simulations. It has been found that recently proposed FSD and SDR based reaction rate closures based on *a-priori* DNS analysis of simple chemistry data perform satisfactorily also for the detailed chemistry case both away from and close to the wall without any adjustment to the model parameters.

## Introduction

Inside combustor chambers, cooling of the walls is necessary, because the burned gas temperature is often higher than the melting point of the combustor material. This cooling has a significant impact on the combustion processes in the near-wall region and on the lifespan of the combustor itself, and this interaction is often referred to as the flame-wall interaction (FWI) [1]. In a Spark Ignition (SI) engine, flame quenching induced by cold walls (e.g. liner and bowl) leads to a formation and an accumulation of unburned hydrocarbons (uHCs), which, in combination with heat losses to the wall, negatively affects the efficiency and pollutant emissions performance. Furthermore, flame propagation in a low-velocity region of the wall boundary layer may lead to flashback from the combustion chamber to the mixing zone in a gas turbine. The increasing demands for lightweight devices, engine downsizing and micro-combustors make FWI an inevitable event in these applications. Therefore, a thorough physical understanding of the FWI mechanism is necessary to develop and design more energy-efficient and environmentally friendly combustion devices. It is difficult to get detailed information of FWI with experimental measurements because of small length-scales and also due to the inherently intermittent nature of these interactions. Direct Numerical Simulation (DNS) offers an opportunity to analyse FWI without recourse to physical approximation, and also to bypass the aforementioned limitations.

In the last few decades, DNS has contributed significantly to the fundamental understanding of turbulent non-reacting and reacting flows, but relatively limited attention has been devoted to the analysis of FWI [2–9]. Poinsot et al. [2] pioneered DNS based analysis of FWI by carrying out two-dimensional simple chemistry simulations of head-on quenching of premixed turbulent flames. Bruneaux et al. [3, 4] analysed side-wall quenching by carrying out three-dimensional incompressible simple chemistry DNS of premixed FWI in a channel flow configuration, and this data in turn was utilised to analyse the influences of the wall on Flame Surface Density (FSD) based reaction rate closure. Alshalaan and Rutland [5, 6] analysed oblique flame quenching by carrying out three-dimensional simple chemistry DNS for the interaction of a V-flame with an isothermal wall, and utilised the resulting data for the analysis of the wall heat flux statistics, and the near-wall behaviours of FSD, and turbulent scalar flux. All the aforementioned DNS analyses [2–6] indicated that the maximum wall heat flux in turbulent flows can assume values greater than the corresponding laminar value due to turbulent convection of flame elements towards the wall. Moreover, stream-wise vortices in a turbulent channel flow push the flame elements towards the wall leading to an increase in the wall heat flux magnitude, whereas convection away from the wall tends to reduce the wall heat flux magnitude [3, 4]. Dabireau et al. [7] analysed premixed FWI for H_2_-O_2_ mixtures based on two-dimensional simulations and demonstrated that the high wall flux magnitudes are obtained at a short time prior to the flame quenching. Gruber et al. [8, 9] carried out a detailed chemistry DNS of FWI in turbulent V-flame and channel flow configurations. They indicated that flashback can be obtained near the wall under some flow conditions, and this behaviour can be affected by combustion instabilities (e.g. Darrieus-Landau instability), which can have significant influences on the near-wall flow dynamics. Recently, Lai and Chakraborty [10] carried out three-dimensional simple chemistry DNS of head-on quenching of statistically planar turbulent premixed flames for different values of global Lewis number (i.e. ratio of thermal diffusivity to mass diffusivity). The findings of Lai and Chakraborty [10] were in agreement with the heat flux and quenching distance statistics obtained from previous two-dimensional simulations [2]. It has been found that the quenching distance for laminar flames increases, whereas the maximum wall heat flux in laminar head-on quenching decreases with decreasing characteristic Lewis number $Le$. However, the maximum wall heat flux in turbulent head-on quenching of statistically planar turbulent flames increases with decreasing characteristic Lewis number and the quenching distance for turbulent sub-unity Lewis number (i.e. $Le<1$) flames has been found to be smaller than the corresponding laminar flame value, whereas the quenching distance for turbulent flames with $Le= 1$, and $Le>1$ remains comparable to their corresponding laminar values. This DNS database was utilised to analyse the statistical behaviours of enstrophy [11], FSD [12] and scalar dissipation rate (SDR) [10, 13, 14] in the near-wall region. Although simple chemistry DNS [2–6, 10–14] provided valuable insights into the physical mechanisms pertinent to turbulent premixed FWI, it is yet to be assessed whether correct quantitative behaviours of wall heat flux and flame quenching distance can be obtained from simple chemistry DNS. Moreover, it has not yet been assessed if the models, which have been proposed based on a-priori analysis of simple chemistry DNS data, remain valid in the presence of detailed chemistry and transport. Furthermore, the implications of flame quenching on the species distribution in the near-wall region, in particular for the intermediate species, are impossible to extract from simple chemistry simulation data. This analysis addresses the aforementioned gaps in the existing literature by carrying out three-dimensional DNS of head-on quenching of a statistically planar turbulent stoichiometric methane-air premixed flame by an isothermal inert wall. The statistics extracted from detailed chemistry DNS regarding wall heat flux, quenching distance, near-wall heat release distribution along with the FSD based mean reaction rate closure in the vicinity of the isothermal inert wall will be compared with those obtained from a corresponding head-on quenching DNS simulation for a generic single-step Arrhenius chemical mechanism with unity Lewis number with same initial normalised turbulence parameters. In summary, the main objectives of this analysis are:

- To compare the wall heat flux and flame quenching distance statistics for detailed and simple chemistry simulations for both laminar and turbulent head-on quenching of premixed flames by isothermal inert walls.
- To demonstrate the near-wall behaviour of intermediate species in head-on quenching of laminar and turbulent premixed flames.
- To compare the model performances in the context of FSD and SDR based mean reaction rate closures for both simple and detailed chemistry simulations.

The rest of the paper will be organised as follows: the mathematical background and numerical implementation pertaining to this work are presented in the next section. Following this, results will be presented and subsequently discussed. The main findings will be summarised and conclusions will be drawn in the final section of this paper.

## Mathematical Background and Numerical Implementation

The detailed chemistry DNS simulation has been conducted using the three-dimensional compressible code SENGA2 [15]. The domain is taken to be a cube of each side equal to 7.65 mm which is discretised by a uniform grid of dimension ${256}^{3}$ ensuring 15 grid points across the thermal flame thickness $\delta _{\text {th}}=(T_{\text {ad}}-T_{0})/\max \left | \nabla \hat {T} \right |_{\mathrm {L}}$ where $\hat {T}$ is the instantaneous dimensional temperature and the subscript ‘L’ refers to the unstrained planar laminar premixed flame value. In SENGA2, the spatial differentiation is carried out using a 10^th^ order central difference scheme for the internal grid points but the order of differentiation gradually decreases to a one-sided 4^th^ order scheme at the non-periodic boundaries. The time advancement is carried out using an explicit low-storage 4^th^ order Runge-Kutta scheme. The negative $x_{1}$-direction is aligned with the mean direction of flame propagation. The left-hand boundary in the $x_{1}$-direction is taken to be an inert isothermal wall which is kept at the unburned gas temperature $T_{0}$, which is taken to be 300 K for this analysis. The boundary opposite to the wall is taken to be partially non-reflecting and is specified using the Navier Stokes Characteristic Boundary Conditions (NSCBC) technique [16]. The transverse directions are taken to be periodic. A skeletal chemical mechanism (involving 16 species and 25 reactions and amongst these 10 reactions are reversible) for atmospheric pressure combustion of methane-air mixture [17] has been considered for detailed chemistry simulations. The thermo-physical properties such as viscosity and thermal conductivity are taken to be functions of temperature, and CHEMKIN [18] polynomials have been used to account for temperature dependence for these physical properties. Furthermore, mixture-averaged transport is adopted for the current analysis, and Soret and Dufour effects are considered in heat and mass transfer. A steady state planar stoichiometric methane-air premixed flame under atmospheric pressure is used for initialising the reacting species and temperature fields. A homogeneous isotropic velocity field, generated using a standard pseudo-spectral method [19] following the Batchelor-Townsend spectrum [20], is used for the initialisation of turbulent fluid motion away from the wall. The initial values of normalised root-mean-square (rms) turbulent velocity fluctuation $u^{\prime }/S_{\mathrm {L}}$, integral length scale to flame thickness ratio $l/\delta _{\text {th}}$, Damköhler number $Da=lS_{\mathrm {L}}/u^{\prime }\delta _{\text {th}}$, and Karlovitz number $Ka=\left (u^{\prime } \left / S_{\mathrm {L}}\right . \right )^{1.5}\left (l \left / \delta _{\text {th}}\right . \right )^{-0.5}$ (where $S_{\mathrm {L}}$, $\delta _{\text {th}}, T_{\text {ad}}$ and $T_{0}$ being the unstrained laminar burning velocity, thermal flame thickness, adiabatic flame temperature and the unburned gas temperature, respectively) away from the wall are summarised in Table 1 along with the heat release parameter $\tau =(T_{\text {ad}}-T_{0})/T_{0} $. The velocity components (i.e. $u_{1}, u_{2}$ and $u_{3})$ are specified to be zero on the wall due to no-slip condition and the diffusive mass fluxes are considered to be zero in the wall normal direction. The initial turbulent flow is allowed to evolve for an integral eddy turn-over time before the reactive simulation is initiated.

**Table 1:** Initial turbulence parameters away from the wall and the value of heat release parameter

Case	Chemical Mechanism	$u^{\prime }/S_{L}$	$l/ \delta _{th}$	$Da$	$Ka$	$\tau $
A	16 species, 25 reactions	7.5	2.5	0.34	13.0	6.0
B	1-step irreversible	7.5	2.5	0.34	13.0	6.0

In order to compare the detailed chemistry simulation results with those obtained from simple chemistry simulation, a three-dimensional DNS for a generic single step irreversible chemistry (i.e. Reactants → Products) has been carried out using the well-known DNS code SENGA [21]. In SENGA, the governing equations of mass, momentum, energy and reaction progress variable *c* are solved in non-dimensional form [21]. The numerical methodologies related to velocity field initialisation, reactive scalar field initilisation, spatial discretisation and time-advancement in SENGA [21] are similar to those used in SENGA2. In SENGA, the thermo-physical properties such as dynamic viscosity, thermal conductivity, and density-weighted mass diffusivity are taken to be constant and independent of temperature. Standard values of Zel’dovich number $\beta =T_{\text {ac}}(T_{\text {ad}}-T_{0})/T_{\text {ad}}^{2}$ (where $T_{\text {ac}}$ is the activation temperature), Prandtl number $Pr$ and ratio of specific heats $\gamma $ (i.e. $\beta = 6.0$, $Pr= 0.7$ and $\gamma = 1.4)$ are used for the simple chemistry simulation where the Lewis numbers of all the species are taken to be unity. For simple chemistry DNS, the domain is taken to be $\left (35.2\delta _{\mathrm {Z}} \right )^{3}$ (where $\delta _{\mathrm {Z}}=\alpha _{\mathrm {T0}}/S_{\mathrm {L}}$ is the Zel’dovich flame thickness with $\alpha _{\mathrm {T0}}$ being the thermal diffusivity for unburned gas) which is discretized using a uniform grid of ${256}^{3}$ ensuring 10 grid points within $\delta _{\text {th}}$. The simulations for head-on quenching have been conducted until the maximum and the minimum wall heat fluxes approach each other, which corresponds to 18 initial eddy turnover times (i.e. $18l/u^{\prime })$ for cases A and B.

For the purpose of evaluating the Reynolds/Favre averaged quantities, the instantaneous quantities of interest are ensemble averaged over $x_{2}-x_{3}$ planes at a given $x_{1}$ location as $x_{2}$ and $x_{3}$ directions are statistically homogeneous directions in this configuration. The Reynolds and Favre averaged quantities are depicted by an overbar and a tilde respectively in this paper. The value of $x^{+}=u_{\tau } {\Delta } x/\nu $ (where $u_{\tau } =\sqrt {\tau _{\mathrm {w}}/\overline {\rho }}$ is the friction velocity, ${\Delta } x$ is the grid spacing and $\nu $ is the kinematic viscosity with $\tau _{\mathrm {w}}$ and $\overline {\rho } $ being the mean wall shear stress and mean gas density respectively) remains smaller than unity during the course of simulation for both cases A and B.

In premixed flames, the scalar field is often characterised in terms of reaction progress variable *c*, and non-dimensional temperature *T*, which can be defined in the following manner:

1 $$ c = \frac{Y_{\mathrm{R0}}-Y_{\mathrm{R}}}{Y_{\mathrm{R0}}-Y_{\text{R} \infty}} \text{and} T = \frac{\hat{T}-T_{0}}{T_{\text{ad}}-T_{0}} $$

where $Y_{\mathrm {R}}$ is the mass fraction of a suitable reactant and the subscripts 0 and $\infty $ indicate values in the unburned reactants and fully burned products, respectively. According to Eq. 1, the reaction progress variable *c* increases monotonically from 0 in unburned reactants to 1 in the fully burned products. For the stoichiometric methane-air flame detailed chemistry simulations, the reaction progress variable *c* is defined based on $\mathrm {C}\mathrm {H}_{4}$ mass fraction which leads to $Y_{\mathrm {R0}}= 0.055$ and $Y_{\text {R}\infty }= 0$. It is also worth noting that alternative definitions of *c* are possible in the context of detailed chemistry simulations by using either an alternative reactant mass fraction (e.g. $\mathrm {O}_{2}$ mass fraction) in Eq. 1 or by using a suitable product mass fraction $Y_{\mathrm {P}}$ in $c=(Y_{\mathrm {P}}-Y_{\mathrm {P0}})/(Y_{\text {P}\infty }-Y_{\mathrm {P0}})$. The implications for different definitions of *c* do not directly affect the analysis conducted here, and thus are not pursued further.

For low Mach number, globally adiabatic, thermo-diffusively neutral flames the non-dimensional temperature *T* can be equated to reaction progress variable *c* but this equality does not hold for head-on quenching due to loss of adibaticity, and also due to different boundary conditions at the wall (i.e. Dirichlet boundary condition applies for temperature for an isothermal wall, whereas a Neumann boundary condition is used for species mass fractions).

## Results & Discussion

**Fig. 1:**
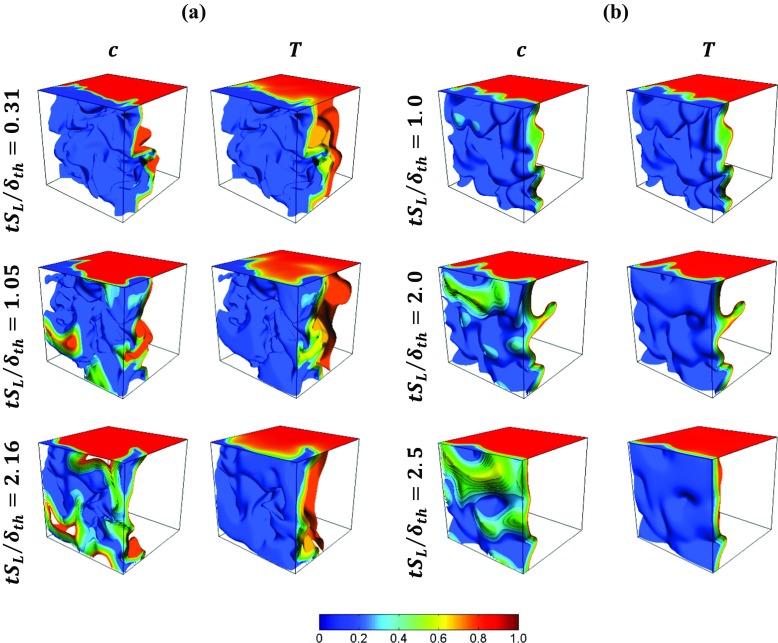
Distributions of *c* (based on $Y_{CH_{4}}$in case A) and non-dimensional temperature *T* at different time instants for **a** detailed chemistry case A, **b** simple chemistry case B. The time instants are different between cases A and B because of the difference in *δ*_*th*_ values

The instantaneous three-dimensional distributions of *c* based on $Y_{\mathrm {C}\mathrm {H}_{4}}$ and non-dimensional temperature *T* at different time instants for the detailed chemistry case (i.e. case A) are shown in Fig. 1a. For the purpose of qualitative comparison, the instantaneous distributions of *c* and *T* at different time instants for simple chemistry are shown in Fig. 1b. It is evident from Fig. 1a that *c* and *T* distributions remain different from each other and the extent of this inequality becomes more prominent as the flame approaches the wall in case A. The extent of this inequality between *c* and *T* remains small when the flame is away from the wall in case B, whereas this inequality remains significant for case A even when the flame is not in the vicinity of the wall. The light species with sub-unity Lewis number (i.e. $Le<1$) such as H and $\mathrm {H}_{2}$ are present within the methane-air flame in the detailed chemistry case, and thus these light species induce local influences of differential diffusion of heat and mass even in a flame which is globally thermo-diffusively neutral in nature. Furthermore, the consumption layer of methane is not coincident with the heat release layer in the stoichiometric methane-air flame, which also contributes to the local inequality between *c* and *T*.

**Fig. 2:**
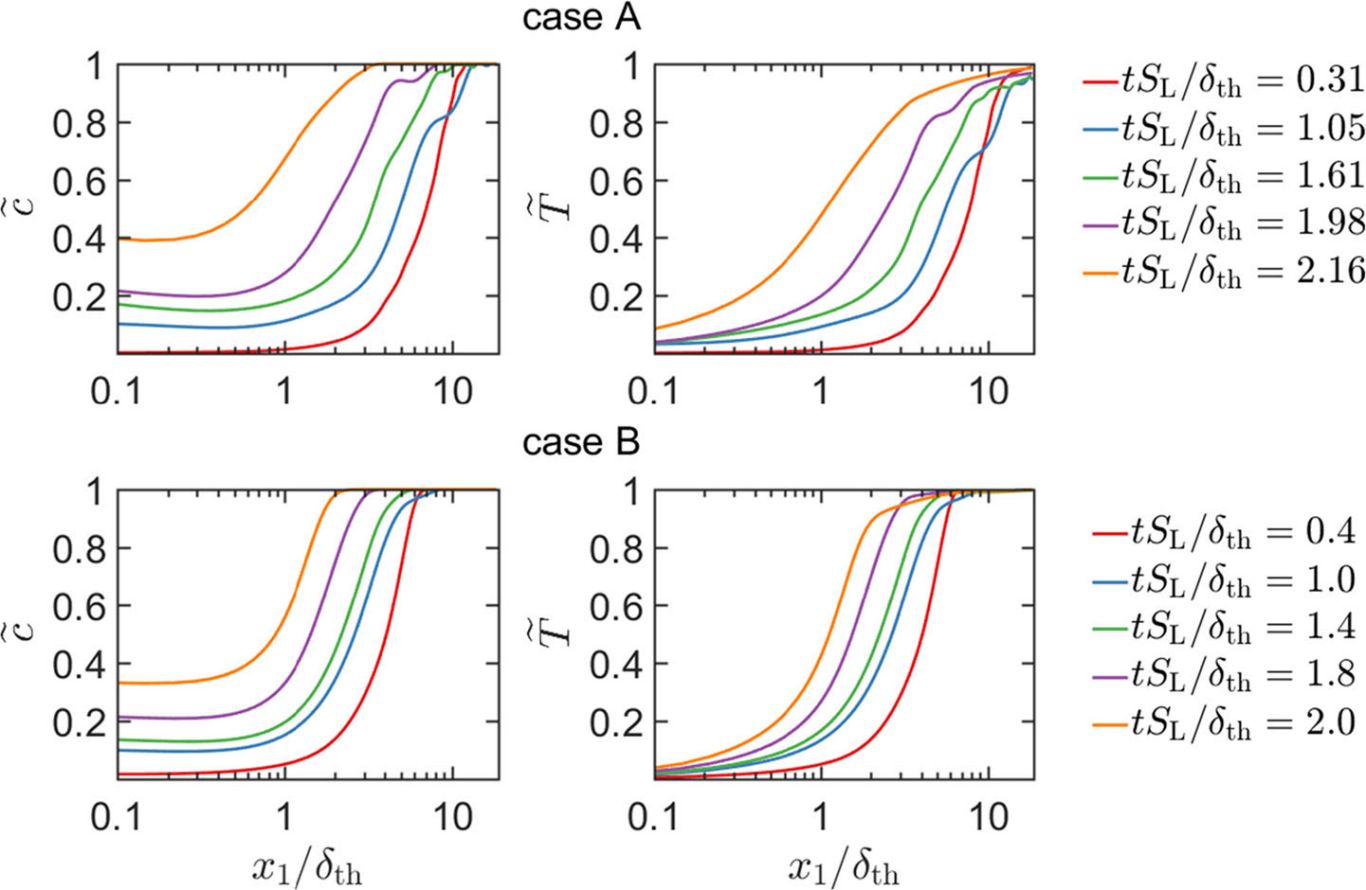
Variations of $\tilde {c}$ and $\tilde {T}$ with *x*_1_/*δ*_th_ at different time instants for case A and case B

**Fig. 3:**
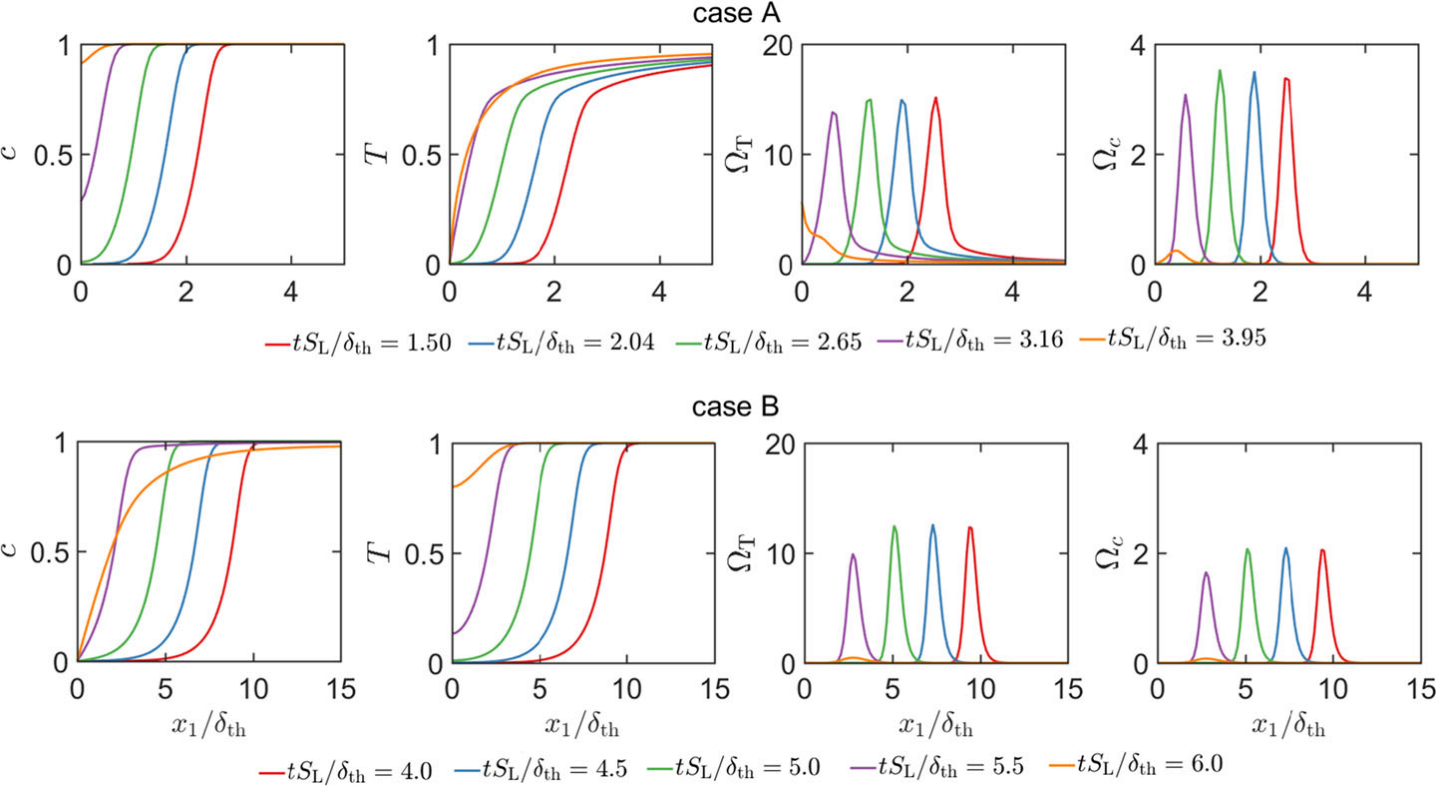
Variations of *c*, *T*, Ω_T_ and ${\Omega }_{c}=\dot {\omega } \times \delta _{\text {th}}/\rho _{0}S_{\mathrm {L}}$ with *x*_1_/*δ*_th_ at different time instants for laminar head-on quenching for both detailed (A) and simple (B) chemistry cases

The inequality between *c* and *T* is also reflected in the behaviour of their Favre-averaged counterparts, which can be seen from the temporal evolutions of $\tilde {c}$ and $\tilde {T}$ for both cases A and B in Fig. 2. In accordance with the observations made from Fig. 1, it can be seen that the inequality between $\tilde {c}$ and $\tilde {T}$ becomes dominant in the vicinity of the wall. The value of $\tilde {c}$ at the wall increases from 0 as the flame interacts with it, whereas $\tilde {T}$ remains zero at the wall. A similar qualitative behaviour has also been observed for *c* and *T* distributions for laminar premixed flames. This can be substantiated from Fig. 3 where the distributions of *c*, *T* and normalised heat release rate $\mathrm {{\Omega }}_{\mathrm {T}}={\dot {\omega }_{\mathrm {T}}\times \delta _{\text {th}}} \left / \left (\rho _{0}S_{\mathrm {L}}C_{\mathrm {p0}}T_{0}\right . \right )$ (where $\dot {\omega }_{\mathrm {T}}=-{\sum }_{i = 1}^{16} {\dot {\omega }_{i}h_{\mathrm {f } i}^{0}} $ is the dimensional heat release term with $\dot {\omega }_{i}C_{\mathrm {p0}}$ and $h_{\mathrm {f} i}^{0}$ being the reaction rate, mixture specific heat at constant pressure in the unburned gas and enthalpy of formation of species *i*, respectively) in the wall normal direction are shown at different time instants for both simple and detailed chemistry cases. The time instants shown in Fig. 3 are different for detailed and simple chemistry cases because $\delta _{\text {th}}$ values for these cases are not the same due to the difference in thermo-chemistry. It can be seen from Fig. 3 that $\mathrm {{\Omega }}_{\mathrm {T}}$ drops significantly once the flame reaches the wall and it vanishes completely once the distance between the flame and the wall becomes smaller than a threshold value in the simple chemistry case B. The reaction rate of progress variable $\dot {\omega } $ vanishes once temperature drops in the vicinity of the wall, which leads to the total disappearance of heat release rate in the near-wall region in the simple chemistry case because the heat release is directly proportional to the reaction rate $\dot {\omega } $ of reaction progress variable in the context of a single-step chemical mechanism. Although the normalised heat release rate $\mathrm {{\Omega }}_{T}$ drops significantly in the vicinity of the wall, $\mathrm {{\Omega }}_{\mathrm {T}}$ does not necessarily vanish at the wall during FWI in the detailed chemistry case even though the normalised reaction rate of reaction progress variable (given by $\mathrm {{\Omega }}_{c}=\dot {\omega } \times \delta _{\text {th}}/\rho _{0}S_{\mathrm {L}}=-\dot {\omega } _{\mathrm {C}\mathrm {H}_{4}} \left / {(Y_{\mathrm {R0}}-Y_{\text {R}\infty })}\right .\times \delta _{\text {th}}/\rho _{0}S_{\mathrm {L}}$ for case A) remains zero at the wall at all times. However, $\mathrm {{\Omega }}_{\mathrm {T}}$ eventually vanishes with further progress of flame quenching even in the detailed chemistry case. It is instructive to look at the species distributions in the detailed chemistry case A in order to explain this behaviour.

**Fig. 4:**
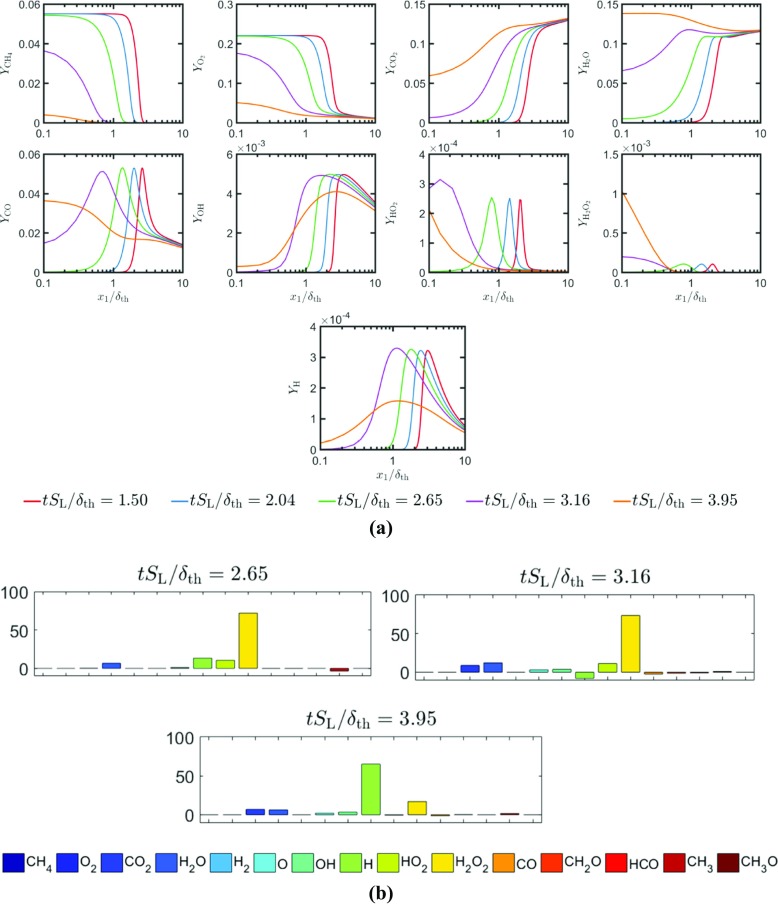
**a** Variation of mass fractions of CH_4_,O_2_,CO_2_,H_2_O,CO,OH,HO_2_H_2_O_2_ and H in the wall normal distance for head-on quenching of a laminar stoichiometric planar premixed flame. **b** Percentage of the overall heat release at the wall arising from different species at different time instants for laminar HOQ according to the detailed chemistry simulation

The temporal evolutions of the distributions of the mass fractions of $\text {CH}_{4},\mathrm {O}_{2},\text {CO}_{2}, \mathrm {H}_{2}\mathrm {O,CO,OH,H}\mathrm {O}_{2}$ and $\mathrm {H}_{2}\mathrm {O}_{2}$ in the wall normal distance for head-on quenching of a laminar stoichiometric methane-air premixed flame are shown in Fig. 4a. It can be seen from Fig. 4a that the mass fractions of $\mathrm {C}\mathrm {H}_{4}$ and $\mathrm {O}_{2}$ both at, and in the vicinity of the wall decrease with time as the flame quenches due to the heat loss through the wall. This drop in ${\text {CH}_{4}}$ mass fraction is reflected in an increase in *c* with the progress of flame quenching. By contrast, the mass fractions of $\text {CO}_{2}$ and $\mathrm {H}_{2}\mathrm {O}$ both at, and in the vicinity of the wall increase with time as the flame quenching advances. This is qualitatively consistent with the observation made from the simple chemistry case in Fig. 3, which indicates that the likelihood of finding products in the vicinity of the wall increases with time during head-on quenching. As long as the flame is away from the wall, the mass fractions of $\text {CO}, \text {OH}$ and $\mathrm {H}$ remain zero in the unburned gas but they assume peak values within the flame before decreasing weakly towards the burned gas side. However, the near-wall behaviour of these species is markedly different. At first the mass fraction of $\text {CO}$ at the wall increases with time during FWI before the concentration of $\text {CO}$ at the wall eventually decreases with time once the flame quenching is in an advanced stage. The mass fractions of $\text {OH}$ and $\mathrm {H}$ remain small at the wall and their values increase in the wall normal direction. It is worth noting that $\text {OH}$ is responsible for $\text {CO}$ oxidation according to $\text {CO}+\text {OH}\rightarrow \text {CO}_{2}\mathrm {+H}$ and this step also gives rise to H which is crucial for chain propagation reactions (e.g. $\mathrm {H+}\mathrm {O}_{2}\mathrm {\rightarrow OH+O}$ and subsequently $\mathrm {O+}\mathrm {H}_{2}\mathrm {O\rightarrow OH+OH})$. The absence of $\text {OH}$ and low temperature in the near-wall region give rise to accumulation (depletion) of $\text {CO}$ (H) in this region during flame quenching because $\text {CO}$ is not oxidised and $\mathrm {H}$ is sufficiently replenished. The diffusion of $\text {CO}$ away from the wall to the interior of the domain eventually leads to an decrease in $\text {CO}$ mass fraction at the wall. It can be seen from Fig. 4a that $\mathrm {H}\mathrm {O}_{2}$ and $\mathrm {H}_{2}\mathrm {O}_{2}$ exhibit significant increase in concentration at the wall during flame-wall interaction. It is worth noting that the reaction steps $\mathrm {O}_{2}\mathrm {+H+M\rightarrow H}\mathrm {O}_{2}\mathrm {+M}$ and $2{\text {HO}}_{2}\mathrm {\rightarrow } \mathrm {H}_{2}\mathrm {O}_{2}\mathrm {+}\mathrm {O}_{2}$ can take place at a low temperature, and these reaction steps are responsible for considerable rise of $\mathrm {H}\mathrm {O}_{2}$ and $\mathrm {H}_{2}\mathrm {O}_{2}$ at the wall. A similar observation was previously reported by Dabireau et al. [7] for premixed FWI of H_2_-O_2_ mixtures based on two-dimensional simulations. These low temperature reactions give rise to heat release rate at the wall during FWI. This can be substantiated from the percentage of heat release (i.e. $\left ({\dot {\omega }_{\alpha } h_{\mathrm {f}\alpha }^{0}} \left / {\sum }_{i = 1}^{16}{\dot {\omega }_{i}h_{\mathrm {f} i}^{0}}\right .\right )\times 100\% $ for species $\alpha )$ at the wall arising from different species at different stages of HOQ, as shown in Fig. 4b.^1^ It can be seen from Fig. 4b that the species involved in the reaction steps $\mathrm {O}_{2}\mathrm {+H+M\rightarrow H}\mathrm {O}_{2}\mathrm {+M}$ and $2{\text {HO}}_{2}\mathrm {\rightarrow } \mathrm {H}_{2}\mathrm {O}_{2}\mathrm {+}\mathrm {O}_{2}$ are the principal contributors to the overall heat release at the wall and the heat release rate contributions from the reactions involving $\mathrm {CO, } \mathrm {H}_{2}\mathrm {O, C}\mathrm {O}_{2}$ are of marginal importance at the wall.

**Fig. 5:**
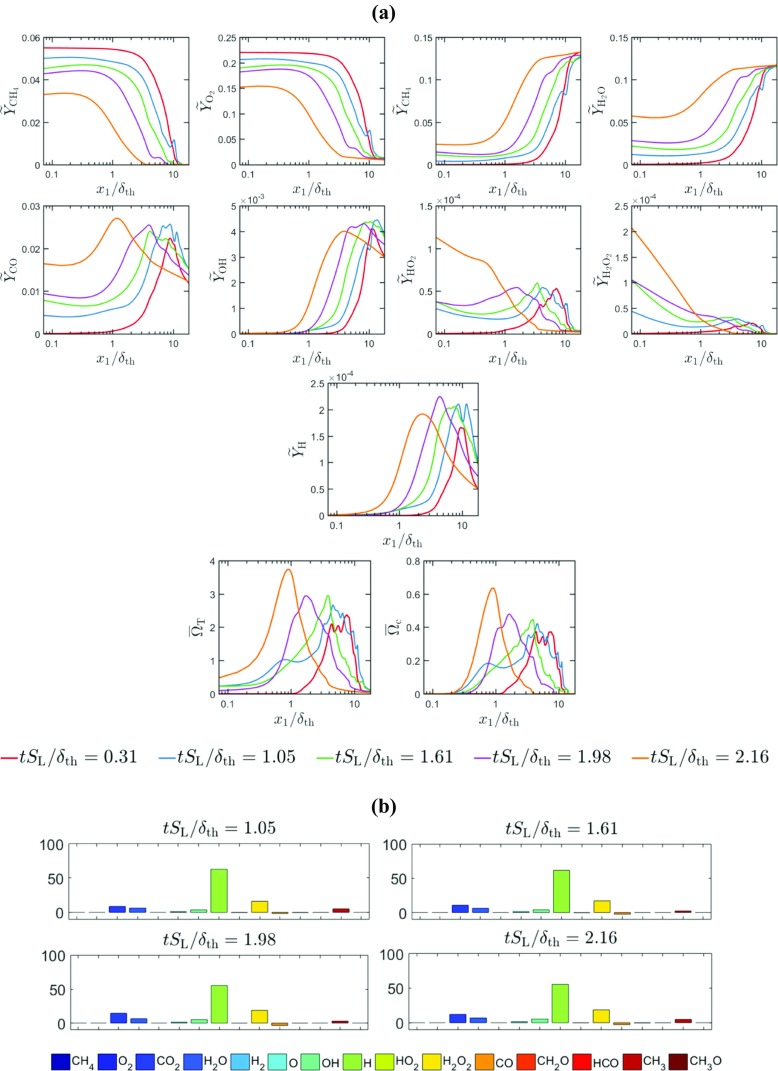
**a** Variation of Favre-averaged mass fractions of CH_4_,O_2_,CO_2_,H_2_O,CO,OH,HO_2_H_2_O_2_, and H; $\bar {{\Omega }}_{T}$ and $\bar {{\Omega }}_{c}$ in the wall normal distance for head-on quenching of a turbulent stoichiometric planar premixed flame (case A). **b** Percentage of the overall heat release at the wall arising from different species at different time instants for turbulent HOQ case A

The temporal evolutions of Favre-averaged mass fractions of $\mathrm {C}\mathrm {H}_{4},\mathrm {O}_{2}\mathrm {,C}\mathrm {O}_{2}\mathrm {, } \mathrm {H}_{2}\mathrm {O,} \mathrm {CO, OH,H}\mathrm {O}_{2}$ and $\mathrm {H}_{2}\mathrm {O}_{2}$, and the Reynolds-averaged heat release rate $\mathrm {{\Omega }}_{\mathrm {T}}$ for turbulent case A are shown in Fig. 5a. The percentages of heat release at the wall arising from different species during the temporal evolution of turbulent HOQ in case A are shown in Fig. 5b. A comparison between Figs. 3, 4 and 5 reveals that the species and heat release distributions in the wall normal distance for the turbulent flame remain qualitatively similar to the corresponding distributions in the case of laminar HOQ. Moreover, similar to the laminar HOQ, the heat release at the wall in the turbulent case A also originates due to reactions involving $\mathrm {H}\mathrm {O}_{2}$ and H_2_O_2_ (i.e. $\mathrm {O}_{2}\mathrm {+H+M\rightarrow H}\mathrm {O}_{2}\mathrm {+M}$ and $2{\text {HO}}_{2}\mathrm {\rightarrow } \mathrm {H}_{2}\mathrm {O}_{2}\mathrm {+}\mathrm {O}_{2})$ and the chemical reactions involving $\mathrm {CO, } \mathrm {H}_{2}\mathrm {O, C}\mathrm {O}_{2}$ do not contribute significantly to the overall heat release at the wall. A comparison between Figs. 4b and 5b reveals that the distributions of ${{(\dot {\omega }}_{\alpha }h_{\mathrm {f } \alpha }^{0}} \left / {\sum }_{i = 1}^{16} {\dot {\omega }_{i}h_{\mathrm {f } i}^{0}}\right .)\times 100\% $ between laminar and turbulent flames are qualitatively different and this may arise because of different reaction pathways in the near wall region during FWI. However, the analysis of this difference is beyond the scope of this paper and will be reported elsewhere in the future.

**Fig. 6:**
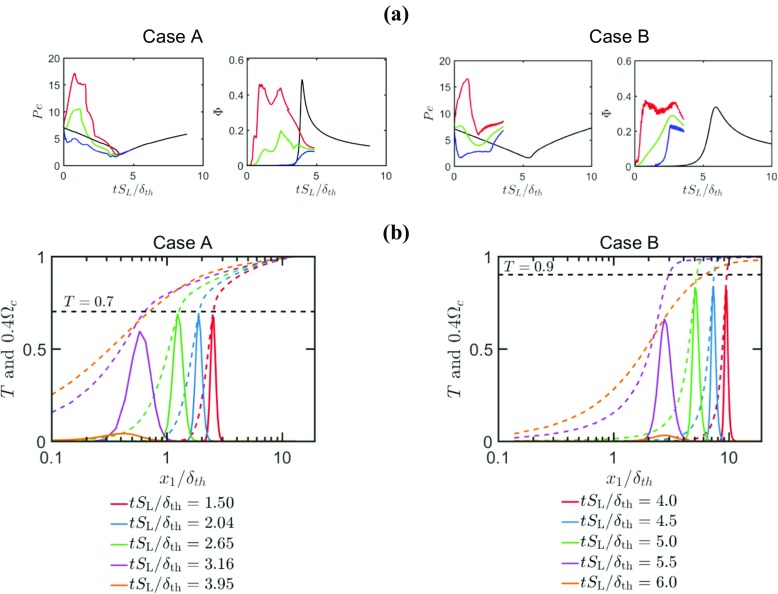
**a** Temporal evolution of maximum, mean and minimum values of wall Peclet number *Pe* (based on *T* = 0.9 isosurface) and non-dimensional wall heat flux Φ along with the corresponding variation obtained for head on quenching of a laminar one-dimensional flame (maximum ; mean ; minimum ; laminar ), The $0.9\left ({\Phi }_{\max }\right )_{\mathrm {L}}$ value is shown by in the plots showing the temporal evolution of Φ. **b** Variations of *T* (broken line) and Ω_*c*_ × 0.4 (solid line) for laminar flame simulation for case A (left) and case B (right)

In head-on quenching, FWI is often characterised with the help of two quantities, which are the wall Peclet number $Pe=X/\delta _{\text {th}}$ and the normalised wall heat flux ${\Phi } =\left | q_{\mathrm {w}} \right |/\left [ \rho _{0}S_{L}C_{P0}\left (T_{\text {ad}}-T_{0} \right ) \right ]$ [2], where *X* is the wall normal distance of $T = 0.9$ isosurface, $q_{\mathrm {w}}=-\lambda (\partial \hat {T}/\partial x_{1})_{\mathrm {w}}$ is the instantaneous wall heat flux with $\lambda $ being the thermal conductivity. The temporal evolutions of the maximum, minimum and mean values of $Pe$ and ${\Phi }$ for cases A and B are shown in Fig. 6a along with the corresponding variation obtained for head on quenching of a laminar one-dimensional flame. The temporal variations of *T* and reaction rate $\dot {\omega } $ of reaction progress variable for laminar flame simulations are also shown in Fig. 6b. It can be seen from Fig. 6a that the flame normal distance of $T = 0.9$ isosurface (or in other words $Pe$) in the laminar flame decreases with time as the flame approaches the the cold wall, and this leads to an increase in wall heat flux ${{\Phi }}$ with time for both simple and detailed chemistry cases. The Peclet number in laminar flame attains the minimum value when the flame quenches (i.e. $T = 0.9$ isosurface is the closest to the wall), which provides the measure of laminar quenching distance. The wall heat flux assumes the peak value in laminar head-on quenching when the minimum value of Peclet number is obtained. Subsequent to flame quenching, the isotherms move away from the cold wall leading to a continuous increase (decrease) in $Pe ({\Phi })$ with time. Based on laminar flame calculation, the minimum value of Peclet number is found to be $Pe_{\min }= 1.6$, whereas the maximum normalised heat flux is found to be $\left ({\Phi }_{\max } \right )_{\mathrm {L}} = 0.34$ for the simple chemistry case. By contrast, detailed chemistry case yields $\left ({\Phi }_{\max } \right )_{\mathrm {L}}= 0.48$ and $\left (Pe_{\min } \right )_{\mathrm {L}}= 2.2$. It is possible to scale ${\Phi }$ as: ${\Phi }\sim {\delta }_{\mathrm {Z}} \left / X\sim 1/Pe\right .$ and thus a higher value of $\left ({\Phi }_{\max } \right )_{\mathrm {L}}$ is expected to be associated with a smaller value of $\left (Pe_{\min } \right )_{\mathrm {L}}$. Nevertheless, the values of $\left ({\Phi }_{\max } \right )_{\mathrm {L}}$ and $\left (Pe_{\min } \right )_{\mathrm {L}}$ for simple and detailed chemistry cases are close to each other, and these values are consistent with previous experimental [22–24] and computational [2] findings at least in the order of magnitude sense. It can be seen from Fig. 6a that for a laminar flame ${\Phi }$ starts to assume non-negligible values when $Pe\approx 4.5$ for both detailed and simple chemistry cases, and this Peclet number provides the measure of the non-dimensional distance at which the flame senses the influence of the wall (i.e. the influence zone) [2]. This implies that the observed HOQ is primarily thermally controlled, and that the chemical mechanism does not have much influence on this influence zone thickness.

It is worth noting that in a freely propagating laminar premixed flame the maximum reaction rate $\dot {\omega } $ of reaction progress variable takes place close to $T\approx 0.85$ for the simple chemistry case (see Fig. 6b). Here the Peclet number is evaluated based on wall normal distance of $T = 0.9$ isosurface following Poinsot et al. [2]. However, the maximum value of $\dot {\omega } $ occurs at a smaller value of *T* (i.e. $T\approx 0.7)$ in the detailed chemistry case than in the corresponding simple chemistry case (see Fig. 6b). Thus, an alternative evaluation of the minimum Peclet number based on the wall normal distance of the isosurface of *T* for which the maximum value of $\dot {\omega } $ occurs will bring the magnitude of $\left (Pe_{\min } \right )_{\mathrm {L}}$ down for the detailed chemistry case (i.e. $\left (Pe_{\min } \right )_{\mathrm {L}}$ based on wall-normal distance of $T\approx 0.7$ turns out to be 1.5) and it will be comparable to $\left (Pe_{\min } \right )_{\mathrm {L}}$ in the simple chemistry case (= 1.6). In the detailed chemistry case, the thermal conductivity and specific heat increase towards the burned gas side due to their temperature dependence, whereas these dependences were not accounted for in the simple chemistry case. This gives rise to the difference in $\left ({\Phi }_{\max } \right )_{\mathrm {L}}$ values between the detailed chemistry and simple chemistry cases. Figure 6a shows that the temporal variations of $Pe$ and ${\Phi } $ in turbulent flames remain qualitatively similar to the corresponding variations in the laminar premixed flame. However, ${\Phi }_{\max }$ in the turbulent cases is found to be comparable to the corresponding laminar flame case for the parameters considered here. Turbulence leads to a broadening of the flame brush due to higher extent of wrinkling, which initiates flame element quenching earlier than the corresponding laminar flame. However, it can be seen from Fig. 6 that the minimum value of wall Peclet number ${Pe}_{\min }$ in turbulent flames remains comparable to the corresponding value in the case of laminar premixed FWI for both simple and detailed chemistry cases, which is consistent with previous simple chemistry DNS based findings [10].

**Fig. 7:**
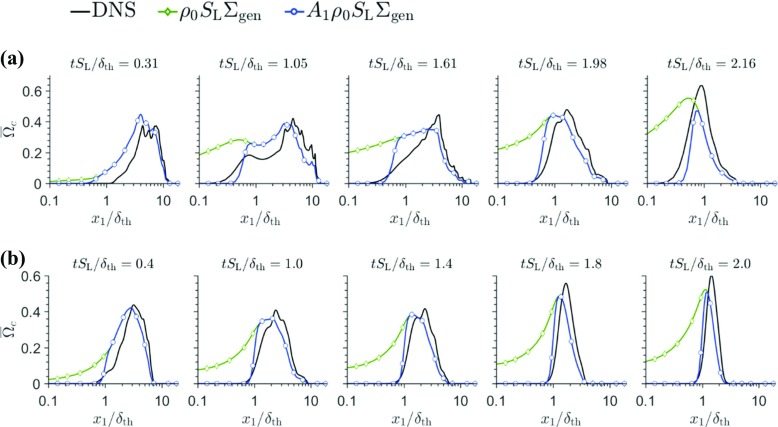
Variations of $\bar {{\Omega }}_{c}=\bar {\dot {\omega } }\times \delta _{\text {th}}/\rho _{0}S_{\mathrm {L}}$ along with the predictions of *ρ*_0_*S*_L_Σ_gen_ × *δ*_th_/*ρ*_0_*S*_L_ and *A*_1_(*ρ*_0_*S*_L_/*Le*)Σ_gen_ × *δ*_th_/*ρ*_0_*S*_L_ with x_1_/δ_th_, for (a-b) cases A and B

The temporal evolutions of mean reaction rate $\overline {\dot {\omega } }$ of reaction progress variable (i.e. $\overline {\dot {\omega }}=-\overline {\dot {\omega }}_{\mathrm {C}\mathrm {H}_{4}}/(Y_{\mathrm {R0}}-Y_{\text {R}\infty })$ in the wall normal direction are shown in Fig. 7 for cases A and B. The mean reaction rate $\overline {\dot {\omega } }$ in turbulent premixed flames is often modelled with the help of generalised FSD ${\Sigma }_{\text {gen}}\mathrm {=}\overline {\vert \nabla c\vert } $ [25] in the following manner:

2 $$ \overline{\dot{\omega} }=\overline{\left( \rho S_{\mathrm{d}} \right)}_{\mathrm{s}}{\Sigma}_{\text{gen}} $$

where $\overline {\left (Q \right )}_{\mathrm {s}}=\overline {Q\left | \mathrm {\nabla } c \right |}/{\Sigma }_{\text {gen}}$ indicates a surface-averaging operation [25] and *S*_d_ = (D*c*/D*t*)/|∇*c*| is the displacement speed. In the context of RANS modelling $\overline {\left (\rho S_{d} \right )}_{s}$ is often modelled as $\overline {\left (\rho S_{\mathrm {d}} \right )}_{\mathrm {s}}\approx \rho _{0}S_{\mathrm {L}}$ [4, 5, 12, 25]. The temporal evolutions of *ρ*_0_*S*_L_Σ_gen_ in the wall normal direction are also shown in Fig. 7. It can be seen from Fig. 7 that $\rho _{0}S_{\mathrm {L}} {\Sigma }_{\text {gen}}$ overpredicts $\overline {\dot {\omega } }$ in the near wall region. It is worthwhile to note that $\overline {\dot {\omega } }$ vanishes completely for $x_{1} \left / \delta _{\text {th}}<\left (P\mathrm {e}_{\min } \right )_{\mathrm {L}}\right . $ but *ρ*_0_*S*_L_Σ_gen_ continues to predict non-zero values in this region for both simple and detailed chemistry cases. This behaviour is consistent with previous simple chemistry DNS based findings [4, 5, 12]. Bruneaux et al. [4] and Alshaalan et al. [5] proposed corrections to the closure $\overline {\dot {\omega }}=\rho _{0}S_{\mathrm {L}} {\Sigma }_{\text {gen}}$ in the near-wall region, which have recently been assessed by Sellmann et al. [12] based on *a-priori* DNS analysis but these models do not adequately predict $\overline {\dot {\omega } }$ in both cases A and B and thus are not shown here.^2^ Sellmann et al. [12] proposed a model expression based on a FSD based $\overline {\dot {\omega } }$ closure, which was found to predict the mean reaction rate for a range of different conditions in terms of turbulence intensity and global Lewis number. The model expression by Sellmann et al. [12] takes the following form for unity Lewis number flames:

3 $$ \overline{\dot{\omega} }=A_{1}\rho_{0}S_{\mathrm{L}}{\Sigma}_{\text{gen}}, \text{ where } A_{1}= 0.5\left[\text{erf}(x_{1} \left/ \delta_{Z}-0.7{\Pi}\right.)+ 1 \right] \text{ and } {\Pi}=\left( {Pe^{\prime}}_{\min} \right)_{\mathrm{L}}\delta_{\text{th}}/\delta_{Z} $$

In Eq. 3, $\left ({Pe^{\prime }}_{\min } \right )_{\mathrm {L}}$ is the Peclet number evaluated based on the wall normal distance of $T = 0.7$ (*T* = 0.9) isosurface for case A (case B) following the previous discussion on the equivalence of minimum Peclet number. It can be seen from Fig. 7 that Eq. 3 satisfactorily captures the variation of $\overline {\dot {\omega } }$ for both simple and detailed chemistry cases both away from and close to the wall. Thus, the reaction rate closure proposed previously based on *a-priori* analysis of simple chemistry DNS data for FWI remains valid also for detailed chemistry simulations of HOQ. The present findings are consistent with recent experimental findings of Jainski et al. [26], which reported that the findings from simple chemistry DNS in the context of FSD based closure remains valid for FWI for a condition with larger value of turbulent Reynolds number.

**Fig. 8:**
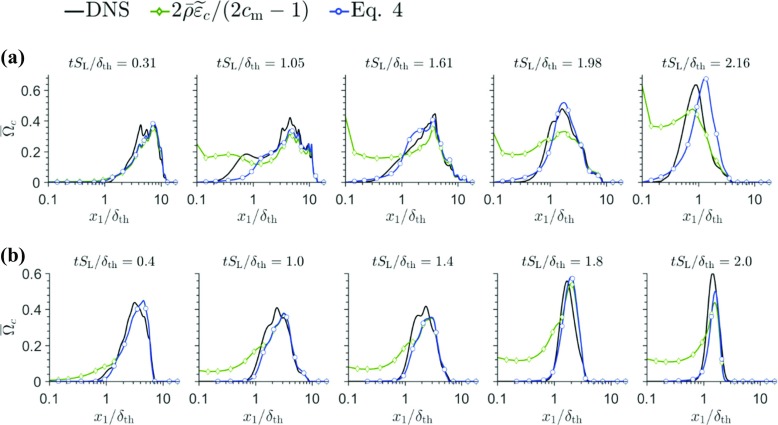
Variations of $\bar {{\Omega }}_{c}=\bar {\dot {\omega } }\times \delta _{\text {th}}/\rho _{0}S_{\mathrm {L}}$ along with the predictions of ${2\bar {\rho } \tilde {\varepsilon _{c}}} \left / \left (2c_{\mathrm {m}}-1 \right )\right .\times \delta _{\text {th}}/\rho _{0}S_{\mathrm {L}}$ and Eq. 4 with x_1_/*δ*_th_ for (a–b) cases

Bray [27] proposed $\overline {\dot {\omega }}={2\overline {\rho } \tilde {\varepsilon _{c}}} \left / \left (2c_{\mathrm {m}}-1 \right )\right .$ where $\tilde {\varepsilon _{c}}=\overline {\rho D\nabla c^{\prime \prime }\cdot \nabla c^{\prime \prime }}\mathrm {/}\overline {\rho } $ is the unresolved SDR, and $c_{\mathrm {m}}={{\int }_{0}^{1}} {\left [ \dot {\omega } c \right ]_{\mathrm {L}}f\left (c \right )\mathrm {d}c} /{{\int }_{0}^{1}} {\left [ \dot {\omega } \right ]_{\mathrm {L}}f\left (c \right )\mathrm {d}c} $ is a thermo-physical parameter (= 0.87 and 0.85 in cases A and B) with $f\left (c \right )$ being the burning-mode probability density function (pdf). The expression $\overline {\dot {\omega } }={2\overline {\rho } \tilde {\varepsilon _{c}}} \left / \left (2c_{\mathrm {m}}-1 \right )\right .$ was derived for $Da\gg 1$ where the pdf of *c* can be approximated by a bimodal distribution with impulses at $c = 0.0$ and $c = 1.0$. Chakraborty and Cant [28] demonstrated that $\overline {\dot {\omega } } ={2\overline {\rho }\tilde {\varepsilon _{c}}} \left / \left (2c_{\mathrm {m}}-1 \right )\right .$ remains valid also for $Da<1$ combustion, provided the flamelet assumption remains valid. The variations of ${2\overline {\rho } \tilde {\varepsilon _{c}}} \left / \left (2c_{\mathrm {m}}-1 \right )\right .\times \delta _{\text {th}}/\rho _{0}S_{\mathrm {L}}$ with normalised wall normal distance $x_{1}/\delta _{\text {th}}$ are shown in Fig. 8 along with the variations of $\overline {{\Omega } }_{c}$ at different time instants. Figure 8 shows that ${2\overline {\rho } \tilde {\varepsilon _{c}}} \left / \left (2c_{\mathrm {m}}-1 \right )\right .$ satisfactorily predicts $\overline {\dot {\omega }}$ for $x_{1}/\delta _{\text {th}}>\left (Pe_{min} \right )_{L}$ before the flame is quenched. However, ${2\overline {\rho } \tilde {\varepsilon _{c}}} \left / \left (2c_{\mathrm {m}}-1 \right )\right .$ deviates significantly from $\overline {\dot {\omega } }$ for $t>(1.05\delta _{th})/S_{\mathrm {L}}$ in cases A and B when the flame interacts with the wall, and starts to quench. Furthermore, Fig. 8 shows that ${2\overline {\rho }\tilde {\varepsilon _{c}}} \left / \left (2c_{\mathrm {m}}-1 \right )\right .$ predicts non-zero values in the near-wall region even when $\overline {\dot {\omega } }$ vanishes due to flame quenching.

**Fig. 9:**
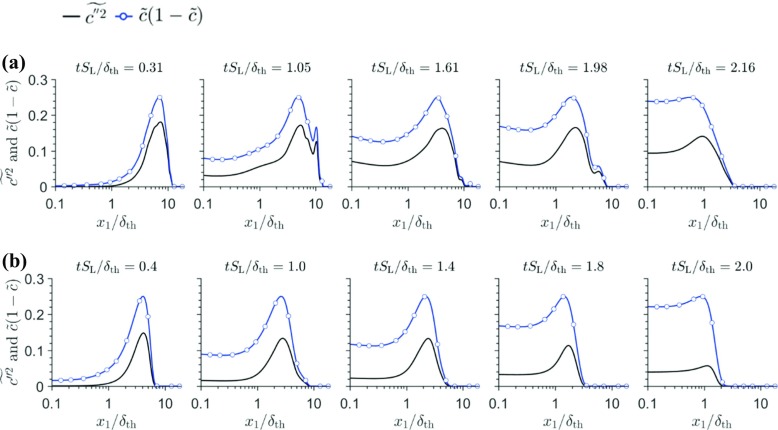
Variations of $\tilde {c^{\prime \prime 2}}$ and $\tilde {c}(1-\tilde {c})$ with *x*_1_/*δ*_th_, for (a–b) cases

**Fig. 10:**
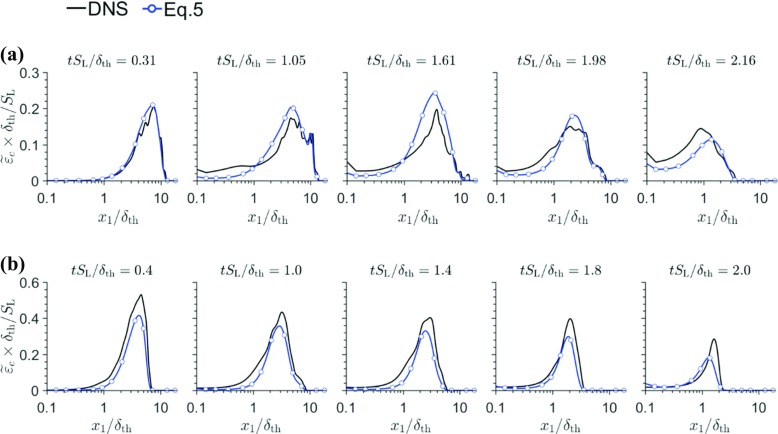
Variations of $\tilde {\varepsilon _{c}}\times \delta _{\text {th}}/S_{\mathrm {L}}$ along with the predictions of Eq. 5 with *x*_1_/*δ*_th_, for (a–b) cases

The variations of $\tilde {c}(1-\tilde {c})$ and $\tilde {c^{\prime \prime 2}}$ with $x_{1}/\delta _{th}$ for cases A and B are shown in Fig. 9. Note that $\widetilde {c^{\prime \prime 2}}=\tilde {c}(1-\tilde {c})$ for a bimodal pdf of *c* with impulses at $c = 0.0$ and $c = 1.0$, which is strictly valid in the limit of $Da\gg 1$. The difference between $\widetilde {c^{\prime \prime 2}}$ and $\tilde {c}(1-\tilde {c})$ provides the measure of departure of the pdf of *c* from a bi-modal distribution. Figure 9 shows that $\widetilde {c^{\prime \prime 2}}$ remains smaller than $\tilde {c}(1-\tilde {c})$ even when the flame is away from the wall, which is representative of $Da<1$ but $\widetilde {c^{\prime \prime 2}}$ almost vanishes when $\tilde {c}(1-\tilde {c})$ assumes non-negligible values during flame quenching. This indicates that the pdf of *c* cannot be considered to be bi-modal and the flamelet assumption is likely to be invalid during flame quenching. As the flamelet assumption is not valid, the model expression $\overline {\dot {\omega } } ={2\overline {\rho } \tilde {\varepsilon _{c}}} \left / \left (2c_{\mathrm {m}}-1 \right )\right .$ ceases to provide satisfactory performance in the near-wall region during flame quenching. Recently, Lai and Chakraborty [10, 13] proposed a $\overline {\dot {\omega } }$ closure in the following manner based on *a-priori* analysis of simple chemistry DNS data:

4 $$ \overline{\dot{\omega} }=\frac{2\overline{\rho} \tilde{\varepsilon_{c}}}{2c_{\mathrm{m}}-1}A_{2}\exp \left( \tilde{c}-\tilde{T} \right)+B_{2}C_{2}\rho_{0}S_{\mathrm{L}} \sqrt{\frac{\tilde{\varepsilon_{c}}}{\tilde{D}}} \exp \left[ -0.5\left( \frac{x_{1}}{\delta_{Z}}-{\Pi} \right)^{2} \right] $$

where $A_{2}= 0.5\left \{ \text {erf}\left [ 3.0\left (x_{1} \left / \delta _{\mathrm {Z}}-{\Pi }\right . \right ) \right ]+ 1 \right \}$, $B_{2}= 0.5\left [ \text {erf}\left (x_{1} \left / \delta _{Z}-{\Pi } \right )\right .+ 1 \right ]$ and $C_{2}= 2.31\text {erf}\left [ 2.6\left (\tilde {c}-\tilde {T} \right ) \right ]$. The quantity, $(\tilde {c}-\tilde {T})$ remains small for $x_{1} \left / \delta _{\text {th}}\gg \right . \left (Pe_{\min }\right )_{\mathrm {L}}$, which leads to $A_{2}\exp \left (\tilde {c}-\tilde {T} \right )\approx 1.0$ and $B_{2}C_{2}= 0$ and thus Eq. 4 reduces to $\overline {\dot {\omega }} ={2\overline {\rho } \tilde {\varepsilon _{c}}} \left / \left (2c_{\mathrm {m}}-1 \right )\right .$ away from the wall. The second term on right hand side of Eq. 4 becomes significant when $(\tilde {c}-\tilde {T})$ assumes large values during flame quenching. Interested readers are referred to [10, 13] for further justification. Figure 9 shows that eq. 4 satisfactorily predicts $\overline {{\Omega }}_{c}$ for both cases without any modification, when $\tilde {\varepsilon _{c}}$ is extracted from DNS data. However, $\tilde {\varepsilon _{c}}$ also needs to be modelled in order to eq. 4 to be useful. Lai and Chakraborty [10, 13] recently modified an existing model for $\tilde {\varepsilon _{c}}$ [28] for HOQ based on *a-priori* analysis of simple chemistry DNS data:

5 $$ \tilde{\varepsilon_{c}}=\frac{A_{\mathrm{\epsilon} }\exp \left[ -1.2\left( \tilde{c}_{\mathrm{w}}-\tilde{T}_{\mathrm{w}} \right)^{3} \right]}{\beta^{\prime}}\left( 2K_{c}^{\ast} \frac{S_{\mathrm{L}}}{\delta_{\text{th}}}+C_{3}\frac{\tilde{\varepsilon} }{\tilde{k}}-\tau C_{4}\frac{S_{\mathrm{L}}}{\delta_{\text{th}}} \right)\tilde{c} \left( 1-\tilde{c} \right) $$

where $\tilde {q}_{\mathrm {w}}$ is the Favre mean value at the wall for a quantity *q* at a given instant of time and $K_{c}^{\ast } ={(\delta }_{\text {th}} \left /{S_{\mathrm {L}})}\right .{{\int }_{0}^{1}} {[\rho \left (D\mathrm {\nabla } c\cdot \mathrm {\nabla } c \right )\mathrm {\nabla \cdot } \vec {u}f\left (c \right )]_{\mathrm {L}}\mathrm {d}c} \left /{{\int }_{0}^{1}}\right . {[\rho \left (D\mathrm {\nabla } c\cdot \mathrm {\nabla } c \right )f\left (c \right )]_{\mathrm {L}}\mathrm {d}c}$ is a thermo-chemical parameter (= 0.87*τ* and 0.78*τ* in cases A and B). In Eq. 5, $A_{\epsilon }= 0.5\left [\text {erf}{\left (x_{1} \left / \delta _{\mathrm {Z}}-{\Pi }\right . \right )+ 1} \right ]$ is a model parameter such that $A_{\epsilon } \exp \left [ -1.2\left (\tilde {c}_{\mathrm {w}}-\tilde {T}_{\mathrm {w}} \right )^{3} \right ]\ne 1$ in the near wall region and reduces to 1.0 away from the wall. In eq. 5, $\beta ^{\prime }= 6.7$, $C_{3}={1.5\sqrt {Ka_{\mathrm {L}}}} \left / \left (1+\sqrt {Ka_{\mathrm {L}}} \right )\right .$ and $C_{4}={1.1} \left / \left (1+Ka_{\mathrm {L}} \right )^{0.4}\right .$ are the model parameters where ${Ka}_{\mathrm {L}}={(\delta }_{\text {th}}\tilde {\varepsilon } /S_{\mathrm {L}}^{3})^{1/2}$ is the local Karlovitz number and $\tilde {\varepsilon } $ is the dissipation rate of turbulent kinetic energy $\tilde {k}$. Interested readers are referred to Refs. [10, 13, 29] for further information on the derivation of Eq. 5. The predictions of Eq. 5 compared to $\tilde {\varepsilon _{c}}\times \delta _{\text {th}}/S_{\mathrm {L}}$ extracted from DNS data are shown in Fig. 10 for cases A and B at different time instants. Figure 10 shows that Eq. 5, which was previously proposed based on simple chemistry DNS data, satisfactorily predicts $\tilde {\varepsilon _{c}}$ both away from and near to the wall for both simple and detailed chemistry cases without any modification to the model parameters.

## Conclusions

The head-on quenching (HOQ) of statistically turbulent planar flames by an isothermal inert wall has been analysed in this study based on three-dimensional compressible DNS simulations for a representative single-step simple chemistry and a multi-step detailed chemical mechanism of methane-air mixture. A skeletal chemical mechanism involving 16 species and 25 reactions for methane-air combustion is used for the purpose of detailed chemistry simulation of HOQ of a stoichiometric methane-air flame. The distributions of reaction progress variable *c* and non-dimensional temperature *T* remain identical to each other away from the wall for simple chemistry simulations but this equality does not hold during head-on quenching. The inequality between *c* defined based on $\mathrm {C}\mathrm {H}_{4}$ mass fraction and non-dimensional temperature *T* holds both away from and close to the wall for detailed chemistry simulations. However, the extent of this inequality becomes particularly prominent in the near-wall region. The value of reaction progress variable *c* and its Favre averaged counterpart $\tilde {c}$ increase at the wall during FWI. In the simple chemistry case, the heat release rate vanishes once the flame reaches a threshold distance away from the wall but a non-zero value of heat release rate can be obtained at the wall during FWI in the detailed chemistry case. Detailed chemistry simulations also revealed that the reaction steps $\mathrm {O}_{2}\mathrm {+H+M\rightarrow H}\mathrm {O}_{2}\mathrm {+M } $ and $2{\text {HO}}_{2}\mathrm {\rightarrow } \mathrm {H}_{2}\mathrm {O}_{2}\mathrm {+}\mathrm {O}_{2}$ can take place at low temperatures which lead to a considerable accumulation of $\mathrm {H}\mathrm {O}_{2}$ and $\mathrm {H}_{2}\mathrm {O}_{2}$ at the wall during HOQ. The aforementioned reaction steps are responsible for heat release at the wall during FWI. The temporal evolutions of wall heat flux and wall Peclet number (i.e. normalised wall-normal distance of $T = 0.9$ isosurface) for both simple and detailed chemistry laminar and turbulent cases have been found to be qualitatively similar. However, small differences have been observed between the numerical values of the maximum normalised wall heat flux magnitude $\left ({\Phi }_{\max } \right )_{\mathrm {L}}$ and the minimum Peclet number $({Pe}_{\min })_{\mathrm {L}}$ based on simple and detailed chemistry laminar head-on quenching calculations. It has been found that the maximum value of the reaction rate of progress variable takes place around *T* = 0.9 for a freely propagating laminar premixed flame under the assumption of simple chemistry but this occurs at around $T = 0.7$ for detailed chemistry. The minimum Peclet number defined based on wall-normal distance of $T = 0.7$ isosurface in the detailed chemistry case is found to be in good agreement with $({Pe}_{\min })_{\mathrm {L}}$ obtained for simple chemistry. The temperature dependence of thermal conductivity and specific heat in the detailed chemistry case leads to higher value of $\left ({\Phi }_{\max } \right )_{\mathrm {L}}$ than the corresponding value for the laminar simple chemistry case. It has been observed that the conventional mean reaction rate closures $\overline {\dot {\omega }} =\rho _{0}S_{\mathrm {L}} {\Sigma }_{\text {gen}}$ and ${2\overline {\rho } \tilde {\varepsilon _{c}}} \left / \left (2c_{\mathrm {m}}-1 \right )\right .$ do not adequately predict the mean reaction rate of reaction progress variable $\overline {\dot {\omega } }$ in the near-wall region for both simple and detailed chemistry simulations. The wall modifications for the FSD and SDR based reaction rate closures based on *a-priori* DNS analysis of simple chemistry DNS data have been found to perform satisfactorily also for the detailed chemistry case without any modifications. Thus, the models, which have been proposed based on *a-priori* analysis of simple chemistry DNS of head-on quenching of turbulent premixed flames, have the potential to be valid even in the presence of detailed chemistry and transport. Although a recent experimental analysis [26] reported that the DNS based findings with moderate Reynolds number remain valid under experimental conditions with much larger Reynolds number values, further investigation with higher value of turbulent Reynolds number will be necessary, which will form the basis of future analyses.

## Footnotes

1 There are some small negative contributions of $\left ({\dot {\omega }_{\alpha } h_{\mathrm {f} \alpha }^{0}} \left / {\sum }_{i = 1}^{16}\right . {\dot {\omega }_{i}h_{\mathrm {f} i}^{0}}\right )\times 100\% $ for some species which is not clear from Fig. 4b. The same is valid for the corresponding turbulent plot in Fig. 5b.

2 Interested readers can see the predictions of mean reaction rate closure proposed in Refs. [4, 5] for simple chemistry cases for different turbulence intensities in Ref. [12].
